# Knowledge and Practices Regarding Chronic Pain After Groin Hernia Surgery: A National Resident Survey in Senegal

**DOI:** 10.3389/jaws.2025.13764

**Published:** 2025-03-13

**Authors:** Abdourahmane Ndong, Adja Coumba Diallo, Adebayo Feranmi Falola, Mamadou Arame Ndiaye, Magatte Faye, Pape Mamadou Faye, Abdou Niasse, Sidy Mouhamed Abdoulaye Fall, Mamadou Cissé, Ibrahima Konaté

**Affiliations:** ^1^ Department of Surgery, Saint-Louis Regional Hospital, Gaston Berger University, Saint-Louis, Senegal; ^2^ Department of Medicine and Surgery, University of Ibadan College of Medicine, Ibadan, Nigeria; ^3^ Department of Surgery, Hôpital Principal, Dakar, Senegal; ^4^ Department of Surgery, Centre Hospitalier National de Dalal Jamm, Dakar, Senegal; ^5^ General Surgery Unit of Cheikh Ahmadoul Khadim Hospital in Touba, Alioune Diop University Of Bambey, Diourbel, Senegal; ^6^ Department of Urology, Centre Hospitalier Regional Heinrich Lübke, Diourbel, Senegal

**Keywords:** chronic pain, hernia, groin, Africa, Senegal

## Abstract

**Background:**

Chronic pain is a significant complication of groin hernia surgery, affecting patients’ quality of life and postoperative recovery. Despite its clinical importance, there is limited research on the knowledge and practice of surgical residents regarding chronic pain management in Senegal. This study aimed to assess the knowledge and practices of surgical residents in Senegal regarding the prevention and management of chronic pain after groin hernia repair.

**Methods:**

A national cross-sectional survey was conducted among surgical residents in Senegal between July 1 and 15 July, 2024. The survey collected data on residents’ demographic characteristics, knowledge of nerve anatomy relevant to groin hernia surgery, and their opinions on the risk factors and treatment strategies for chronic pain.

**Results:**

A total of 74 residents participated in the survey. There were specializing in general surgery (59.5%) and urology (40.5%). While 89.2% of the respondents recognized chronic pain as a complication of hernia surgery, only 9.5% (n = 7) demonstrated a comprehensive understanding of both the relevant nerve anatomy and the definition of chronic pain. Opinions regarding the role of mesh and laparoscopic surgery in increasing the risk of chronic pain were mixed. Additionally, 47.3% of the participants disagreed that surgery may be required to manage chronic pain, reflecting gaps in training.

**Conclusion:**

This study identified significant knowledge gaps among surgical residents in Senegal regarding the prevention and management of chronic pain after groin hernia surgery. There is a need for enhanced training programs that focus on chronic pain management, including nerve identification and evidence-based treatment strategies, to improve patient outcomes.

## Introduction

Inguinal hernia repair is a frequently performed surgical procedure, with millions of cases being treated globally each year [1]. Chronic postoperative pain remains a significant complication of groin hernia repair, influencing patients’ postoperative hospital stay and quality of life [2]. Several factors can increase the risk of chronic pain after inguinal hernia surgery, including acute postoperative complications such as seroma, hematoma, and infection, which are primarily associated with the quality of surgery [3]. However, surgical advancements such as minimally invasive groin hernia repair (laparoscopy and robotics) have led to a reduced incidence of postoperative pain and faster patient recovery [4]. These minimal access techniques, however, are yet to be implemented in the majority of African settings [5].

Chronic pain, defined as pain persisting for more than 3 months following surgery, is a complex and multifactorial postoperative complication that is particularly prevalent following groin hernia repair [6–8]. The incidence of chronic pain after inguinal hernia surgery varies widely in the literature depending on the methods of pain assessment and length of follow-up [1, 9]. The cause of chronic pain after hernia repair is multifactorial involving surgical techniques such as repair under tension, tight closure of the inguinal ring, nerve damage caused by transection or suture entrapment, mesh-related complications, or a combination of these factors [1, 10, 11]. Surgical techniques, including the use of mesh and the fixation method, have been shown to influence the likelihood of developing chronic pain [1, 11, 12]. For example, while lightweight meshes and specific fixation methods, such as glue, have been associated with reduced pain in some studies, these findings are not universally consistent [13, 14]. Additionally, the identification and management of nerves during surgery, such as the ilioinguinal, iliohypogastric, and genital branches of the genitofemoral nerve, can minimize the risk of chronic pain [15].

The overall pooled prevalence of chronic pain following groin hernia surgery in Africa is 2.7% [16], whereas in Senegal, an incidence of 9.2% has been recorded, with most cases (8.2%) being mild [3]. However, the burden of chronic pain following groin hernia surgery in Africa may be underrecognized and undertreated, possibly due to limited training and resources for the management of chronic pain in the postoperative setting [16, 17].

Understanding the knowledge and practices of surgical residents is crucial for improving patient outcomes and addressing public health challenges posed by chronic postoperative pain [18, 19]. This study aimed to assess the knowledge and practices regarding chronic pain after groin hernia surgery among surgical residents in Senegal.

## Methodology

The Checklist for Reporting Results of Internet E-Surveys (CHERRIES) guidelines were followed for this study [20].

### Design

This study was a cross-sectional national survey. The study period was from July 1 to July 15, 2024.

### Population and Sampling

The target population included all surgical residents currently undergoing training in Senegal. Only specialties dealing with groin hernias were included (general surgery and urology). Contact information (phone numbers and/or email addresses) was obtained from the Senegalese resident associations. Four cycles of email and messages were sent to increase the response rate.

### Study Setting

This study was conducted in Senegal, a West African country, with an estimated population of 18,032,473 in 2023. There are five public universities in Senegal, of which 3 have surgical residency programs.

### Survey Tool and Studied Parameters

A structured questionnaire was designed to collect data on the knowledge and practices regarding chronic pain after groin hernia surgery.

The questionnaire was pre-tested to ensure clarity and relevance and was designed to be concise and user-friendly for ease of completion. The questions focused on the fundamental knowledge that residents should have regarding chronic pain prevention and treatment. The definitions and questions were based on the International HerniaSurge guidelines for groin hernia management, as well as the definition of chronic pain provided by Andresen et al. in their study on the management of chronic pain after hernia repair [6].

The questions were divided into three sections.

• Section I: Demographic data

The collected data included specialty, university, year of specialization, sex, and age.

• Section II: Knowledge on chronic pain

This section focused on assessing the residents’ knowledge of chronic pain related to groin hernia, their understanding of chronic pain following hernia repair, and the risks of nerve injury during open inguinal surgery. The questions included:

• The definition of chronic pain after hernia repair, specifying a timeframe of 3 months for considering pain as chronic [6].
• Identification of the three nerves likely to be damaged during open groinsurgery (ilioinguinal, genitofemoral, and iliohypogastric). To avoid inconsistencies, four false nerve responses were included in the questionnaire to prevent random positive answers.

Good knowledge was defined as the ability to correctly identify the three main nerves and understand the timeframe after surgery when pain should be considered chronic.

• Section III: Opinions on Risk Factors and Treatment

This section addressed residents’ opinions regarding the risk factors and treatment strategies to prevent or manage chronic pain after groin hernia surgery. The questions included:

• Opinions regarding chronic pain after groin hernia repair, including its classification as a complication, its relationship with mesh treatments and laparoscopic procedures, and the potential need for surgical revision
• Views on systematically cutting or identifying major nerves during open surgery to prevent chronic pain.

### Data Analysis

Qualitative variables were described in terms of frequency and proportion, whereas quantitative variables were presented as means with their standard deviations. Graphs were generated using Datawrapper.de [21].

### Consent

All participants were asked for consent before participation, and all data were anonymized.

### Ethical Considerations

The study protocol was approved by the Institutional Review Board. Participation was voluntary, and the respondents could withdraw at any time. To ensure anonymity, no personal information was collected.

### Statistical Analysis

Data were analyzed using the R software. Descriptive statistics were used to summarize the demographic data and responses.

## Results

### Demographic Data

A total of 74 surgical residents participated in the national survey (Table 1). The majority of the respondents were from general surgery (59.5%, n = 44), with the remainder from urology (40.5%, n = 30). Participants were predominantly from Cheikh Anta Diop University (51.4%, n = 38), followed by Gaston Berger University (35.1%, n = 26) and the University of Thiès (13.5%, n = 10).

**TABLE 1:** Demographics data of respondents (n = 74).

Variable		N (%) Mean ± sd
Sex (%)	Female	10 (13.5)
	Male	64 (86.5)
Age		31.3 ± 3.5
Speciality (%)	General surgery	44 (59.5)
	Urology	30 (40.5)
University (%)	Cheikh Anta Diop University	38 (51.4)
	Gaston Berger University	26 (35.1)
	University of Thiès	10 (13.5)
Year of residency (%)	1st year	19 (25.7)
	2nd year	15 (20.3)
	3rd year	15 (20.3)
	4th year	14 (18.9)
	5th year	11 (14.9)

The distribution across residency years showed a slight predominance of first-year residents (25.7%, n = 19). The sample was predominantly male (86.5%, n = 64), with a mean age of 31.3 ± 3.5 years. The demographics are detailed in Table 1.

### Level of Knowledge

**FIGURE 1:**
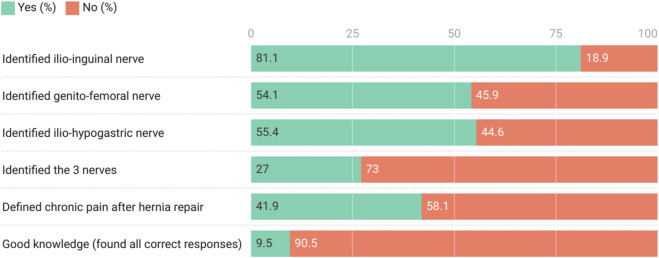
Level of knowledge of respondents (n = 74).

Regarding knowledge of nerve injury risks during open inguinal surgery, the majority of residents correctly identified the ilioinguinal, genitofemoral, and iliohypogastric nerves as susceptible to damage. Only 27% (n = 20) of participants correctly identified the three nerves. Chronic pain after hernia repair was correctly defined in 41.9% (n = 31). Only 9.5% (n = 7) were considered to have a good level of knowledge by identifying all three nerves and correctly defining chronic pain (Figure 1).

### Opinions on Risk Factors and Treatment

A significant majority of residents (89.2%, n = 66) disagreed or strongly disagreed with the statement that chronic pain after groin hernia repair is a normal occurrence rather than a complication. Opinions were divided regarding whether mesh treatment increased the risk of chronic pain, with 73% (n = 54) disagreeing or strongly disagreeing.

Regarding laparoscopic procedures, there was no consensus on whether they increased the risk of chronic pain, with responses being fairly evenly distributed between disagreement (33.8%, n = 25), neutrality (32.4%, n = 24), and strong disagreement (32.4%, n = 24).

The majority of residents (83.8%, n = 62) disagreed or strongly disagreed with the notion of systematically cutting the main nerves during open surgery to avoid chronic pain. Conversely, there was strong agreement (81%, n = 60) that major nerves must be systematically identified during open surgery to avoid chronic pain.

**FIGURE 2:**
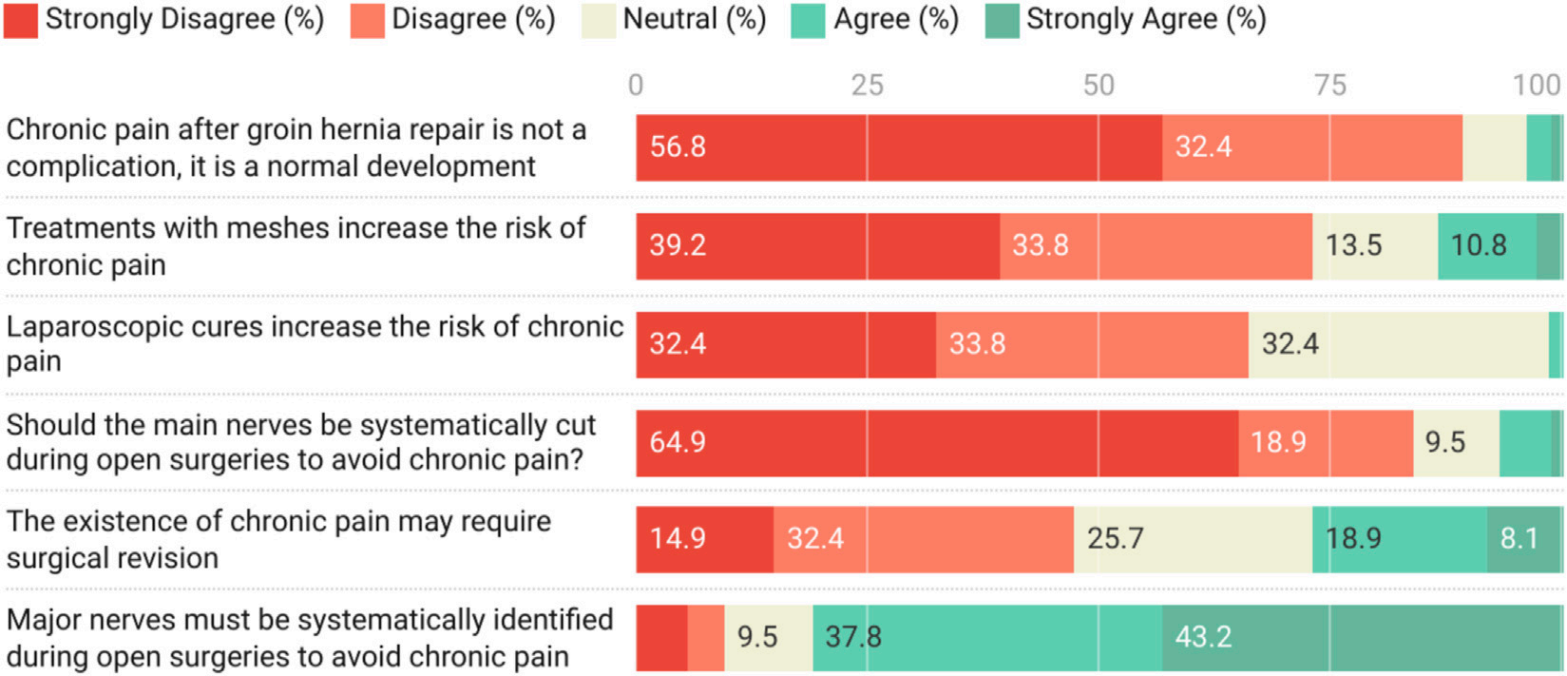
Opinions on Risk Factors and Treatment about chronic pain after groin hernia surgery (n = 74).

Opinions were mixed regarding whether the existence of chronic pain may require surgical revision, with 47.3% (n = 35) disagreeing or strongly disagreeing, 25.7% (n = 19) neutral, and 27% (n = 20) agreeing or strongly agreeing (Figure 2).

## Discussion

Chronic pain is a major complication following groin hernia surgery, affecting 10%–20% of patients globally, causing significant disruption of daily activities in 0.5%–6% [1]. This survey assessed the knowledge of general surgery and urology residents in Senegal regarding chronic pain after inguinal hernia repair.

Although the majority (89.2%, n = 66) of the respondents acknowledged that chronic pain after groin hernia repair is a complication, only 9.5% (n = 7) of the participants were considered to have a good level of knowledge about it, as they correctly identified the three nerves and accurately defined chronic pain.

Knowledge of nerve anatomy in the inguinal region is fundamental for the prevention and management of chronic pain [22]. Despite the existence of several anatomical variations in this innervation, understanding the classical arrangement of this anatomy is a cornerstone that should be included in surgical training of surgeons to ensure proper management of chronic pain.

The ilioinguinal, iliohypogastric, and genitofemoral nerves were correctly identified as susceptible to damage by 81.1%, 54.1%, and 55.4% of study participants, respectively. When present, the ilioinguinal nerve is considered at risk when it is compromised in the early stages of surgery and when the nerve or its medial or lateral branches obscure the surgical field [22, 23]. The genital branch of the genitofemoral nerve is at risk when a thick cremaster muscle must be divided, as it may be included in a ligature of the muscle. Previous studies have confirmed the importance of nerve identification. In their case series, Smeds et al. identified the ilioinguinal nerve in 73% of patients, iliohypogastric nerve in 65%, and genital branch of the genitofemoral nerve in 14% [23]. Studies have shown that identification of all three nerves is difficult in open hernia surgery [24]. Non-identification of any combination of nerves is associated with significant chronic pain in the postoperative period [23, 25]. The ilioinguinal nerve, especially when not identified, has been shown to be associated with poorer outcome [22, 23, 25]. For this reason, significant attention has been paid to the intraoperative identification and handling of the ilioinguinal nerve during inguinal hernia repair [23]. The preservation or resection of nerves identified during inguinal surgery is still an ongoing debate [2]. While some studies have reported better outcomes, including significantly reduced postoperative chronic pain when the ilioinguinal nerve was successfully identified and resected [26, 27], other studies have reported that resection of the ilioinguinal nerve did not significantly reduce postoperative chronic pain [28, 29]. The majority (83.3%) of the surgical residents in our study disagreed with preemptive resection of the identified nerves to prevent chronic pain.

In terms of risk factors, opinions of the respondents regarding whether mesh treatment or laparoscopy increased the risk of chronic pain were mixed. Although recent updates in international guidelines on hernia surgery suggest that these two factors do not seem to be associated with an increased risk of chronic pain, some practitionners still have different opinions [30]. This suggests that training on chronic pain management and research is not uniformly implemented in our context. Moreover, a systematic review of groin hernias in Africa revealed that although the rate of chronic pain was low (2.7%), this outcome has not been systematically assessed in previous studies [16]. When assessed, the methods and periods of evaluation are often inadequate or unspecified [31].

Furthermore, up to 47.3% (n = 35) of the participants disagreed that surgery might be required for the management of chronic pain, indicating a gap in surgical training related to this issue. A specific training program for chronic pain should be developed to raise awareness regarding its assessment and treatment. Future studies on hernia surgery should also systematically evaluate chronic pain to determine its exact burden and best practices for its management.

### Strengths

This study included all surgical specialties and residency levels involved in hernia surgery, representing a significant proportion of the target population. To the best of our knowledge, this is the first study to evaluate training in chronic pain after hernia surgery in our context. Due to cultural barriers and the limited availability of cadaveric specimens in our context, improving training through low-cost simulation models and virtual reality (VR) tools, combined with online training modules, could provide accessible and standardised knowledge, particularly in the prevention and management of chronic postoperative pain. This study could also contribute to defining best practices or developing local guidelines for the management and prevention of chronic pain after groin hernia surgery in Senegal.

### Limitations

The survey relied on self-reported knowledge and practices, which may be influenced by bias, as residents might report choices that they believe to be ideal rather than their actual practices. Besides, we were unable to calculate the exact response rate, as we had access to the contact information of all residents regardless of their specialty. However, only urology and general surgery residents responded to the questionnaire. Moreover, the survey did not assess practical skills or decision-making abilities in real-world clinical scenarios like how participants manage chronic inguinal pain after surgery. We acknowledge that our sample size was relatively small, which limited the possibility of conducting robust subgroup analyses, such as comparing responses across different years of residency. Future studies should aim to increase participant numbers, potentially through multi-country studies, to enhance the statistical power and allow for more detailed comparisons. In addition, the study was limited to surgical residents in Senegal, which may restrict the generalizability of the findings to other settings with different medical training systems.

## Conclusion

This study revealed notable gaps in the knowledge and practices of surgical residents in Senegal concerning the prevention and management of chronic pain after groin hernia surgery, indicating areas that could benefit from improvements in surgical education and training programs. Although most residents recognized chronic pain as a complication, only a small proportion demonstrated comprehensive knowledge of relevant nerve anatomy and pain management strategies. These findings emphasize the need for enhanced training programs that focus on chronic pain management, including the identification of at-risk nerves during surgery and application of evidence-based treatment strategies. Incorporating systematic assessments of chronic pain into surgical education and practice is essential to improve patient outcomes and reduce the burden of chronic postoperative pain. Future studies should explore the long-term effects of these educational interventions.

## Data Availability

The raw data supporting the conclusions of this article will be made available by the authors, without undue reservation.
